# Supplementary material for: Patient-Derived Organoids of Cholangiocarcinoma
Source: Int J Mol Sci. 2021 Aug 12;22(16):8675. doi: 10.3390/ijms22168675 (PMC8395494; doi:10.3390/ijms22168675)
Supplement: Supplementary file 1 [file ijms-22-08675-s001.zip › IJMS, Appendix A CM_ssc_LZ.pdf]

International Journal of *Molecular Sciences*

MDPI

Article

Patient-Derived Organoids of Cholangiocarcinoma

Christopher F. Maier[1,2,†], Lei Zhu[1,2,†], Lahiri K. Nanduri[3,†,§], Daniel Kühn[3,‡], Susan Kochall[3], May-Linn Thepkaysone[3], Doreen William[4,5], Konrad Grützmann[4,5], Barbara Klink[4,5,6], Johannes Betge[7,8], Jürgen Weitz[3], Nuh N. Rahbari[2], Christoph Reißfelder[2] and Sebastian Schölch[1,2,*]

1. Supplementary Methods

1.1. Organoid culture medium

Organoid culture medium consisted of 50 % Wnt3a (prepared as described earlier [1]), 24.6% AdDMEM/F12 (Invitrogen), 10% R-spondin (R&D Systems), 10 %Noggin (Thermo Fisher Scientific), 2% B27-Supplement 50x (Life Technologies), 1% Nicotinamide (10 mM) and 1% Insulin-Transferrin-Selenium (ITS) 100x (both from Sigma Aldrich), 0.4% A83.01 (2µM) (Tocris), 0.2% N-Acetyl-L-Cysteine (1 mM) (Sigma Aldrich), 0.2% FGF10 (200 ng/ml) (PeproTech), 0.2% Primocin (Invivogen), 0.1% Y-27632 (Sigma Aldrich), 0.1% EGF (1:10, PeproTech), 0.1% Gastrin 1 µM (Sigma Aldrich) and 0.1% Forskolin (Tocris).

1.2. In vitro treatment assays

Organoids were collected, transferred to a 96 well plate in 50 µl matrigel and overlaid with 100 µl organoid medium. Drugs were prediluted with organoid medium and added 24 h after seeding. Treatment was performed using gemcitabine (0.01, 0.1, 10 and 100 µM), sorafenib (1, 5, 10 and 25 µM), cisplatin (1, 10, 50 and 100 µM) and doxorubicin (1, 10, 100 and 500 µM). 2D cell lines were treated with gemcitabine (0.01, 0.1, 10 and 100 µM) and sorafenib (1, 10, 20 and 30 µM). About 10^4 cells/well were seeded on a 96 well plate. After 24 h of incubation, prediluted drugs were added.

Cell viability was measured after 72-96 h incubation using the PrestoBlue Cell Viability Reagent (Invitrogen). PrestoBlue was added to the cells and incubated at 37 °C for 3 hours. Fluorescence was measured by Varioskan LUX multimode microplate reader (Thermo Scientific), an excitation wavelength of 560 nm and an emission of 590 nm.

1.3. Xenograft treatment

The tumor from a subcutaneous P68 organoid xenograft mouse was resected and minced into small fragments (1x1mm). These fragments were again subcutaneously reimplanted into NSG™ mice (8-12 weeks old). After newly formed tumors gained a volume of 62.5 mm^3, treatment with gemcitabine was started (5 mice). The intraperitoneal application was performed twice a week with a concentration of 100 mg/kg body weight. Glucose solution was used as a control (5 mice). Subcutaneous tumors were measured regularly with caliper and tumor volume was calculated using the following formula: $V = (Width^2 \times Length)/2$.

1.4. Organoid histology preparation

To prepare the histology checking of organoids, we collected 5 complete wells of each organoid line, fixed with 4% paraformaldehyde for 30 min at room temperature (RT), and washed twice with phosphate-buffered saline. Then organoids were incubated in ethanol with increasing concentrations (25%, 50% and 70%) for 15 min in each case at RT. Obtained organoid pellets were resuspended in 96% ethanol with 1% eosin and incubated

Citation: Maier, C.F.; Zhu, L.; Nanduri, L.K.; Kühn, D.; Kochall, S.; Thepkaysone, M.-L.; William, D.; Grützmann, K.; Klink, B.; Betge, J.; et al. Patient-Derived Organoids of Cholangiocarcinoma. *Int. J. Mol. Sci.* **2021**, *22*, 8675. https://doi.org/10.3390/ijms22168675

Academic Editor: Daniela Taverna

Received: 22 June 2021
Accepted: 7 August 2021
Published: 12 August 2021

Publisher's Note: MDPI stays neutral with regard to jurisdictional claims in published maps and institutional affiliations.

Copyright: © 2021 by the authors. Submitted for possible open access publication under the terms and conditions of the Creative Commons Attribution (CC BY) license (http://creativecommons.org/licenses/by/4.0/).

for 30 min at RT. Afterward, organoids were incubated three times in pure ethanol and then three times in 1-butanol (both from Merck) for 30 min every time at RT. Finally, cells were embedded into paraffin.

1.5. Next-Generation Sequencing

Next-generation sequencing was performed in the Core Unit for Molecular Tumor Diagnostics of the National Center for Tumor Diseases (NCT) Dresden. The nucleic acid of samples was isolated using Qiagen RNeasy kits and QIAamp DNA kits according to the manufacturer's protocols. RNA and DNA quality was confirmed with a Fragment analyzer and QuBit. The TruSeq Stranded mRNA kit was used for RNA sequencing and enrichment for whole-exome sequencing was performed using the Nextera Rapid Capture Exome protocol (both Illumina). Sequencing was conducted on a Nextseq 500 platform (Illumina) with an average of 12 million total reads per sample for RNA sequencing and a median coverage of 150 reads for exome sequencing.

1.6. RNA-Seq read mapping and differential expression analysis

Paired-end reads were trimmed for quality and sequence adapters using trimmomatic [2]. Reads were aligned against the phase II reference of the 1000 Genomes Project, including decoy sequences d5 (hs37d5) using STAR [3] in a two-pass mapping mode. Read counts of all annotated genes (Gencode GRCh37.p13) were extracted from the alignments using the featureCounts method of the Rsubread package [4]. rRNA, immunoglobulin and T-cell receptor genes, and genes with 0 counts for all samples were discarded. DESeq2 [5] was applied to find differentially expressed genes with standard parameters. Only genes with multiple testing adjusted p-values (padj from DESeq2) < 0.01 and $|\log_2(\text{Fold Change, FC})| > 1$ were considered significant.

1.7. Gene ontology and pathway analysis of transcriptomic data

The functional enrichment analysis was completed by the clusterProfiler software package [6], which consists of the functional interpretations of Gene Ontology (GO) [7] and the Kyoto Encyclopedia of Genes and Genomes (KEGG) [8]. This study applied the Benjamini–Hochberg method for the false discovery rate. Only enriched terms with minimum 3 genes annotated, p-value < 0.01 and q value < 0.05 were identified as significantly enriched.

1.8. Gene set enrichment analysis (GSEA)

GSEA was conducted on Gene set enrichment analysis (GSEA) 3.0 (Broad Institute, Inc., Massachusetts Institute of Technology). For P68 specific signatures, this study chose the features with $\log_2$Fold Change ≥ 4 and p-value $< 1 \times 10^{-4}$ in the P68 tumor compared to the healthy tissue. This study set 1000 times of permution, Sinal2Noise metric, and phenotype comparison for the analysis.

1.9. Whole-exome sequencing analysis

In exome sequencing, reads were processed using CLC biomedical workbench v5.0 software (Qiagen). Sequencing reads were aligned to the human reference genome GRCh37 (hg19) using two different gap cost models (linear and affine) to enhance correct alignments of reads with larger insertions or deletions (InDels) as well as small variants (SNVs, MNVs, small InDels). Somatic variants were identified by subtracting variants in the alignment of a matched blood control from tumor and organoid data. Variants with a frequency below 10%, fewer than three supporting reads or coverage below 10 reads were

discarded. Copy number variations (CNVs) were called using cnvkit [9] using circular binary segmentation and fix for regional coverage and GC content bias correction.

2. Supplementary Figures

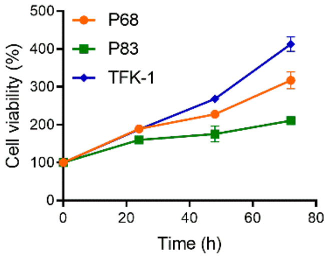

Figure S1. Proliferation curves of two-dimensional cell lines.

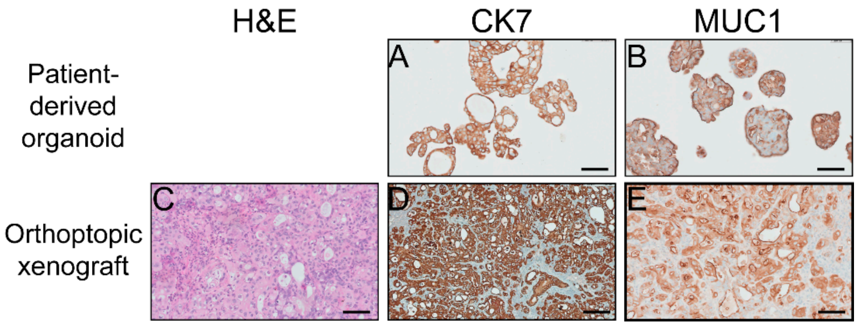

Figure S2. Histologic assessment of P68 organoids and orthotopic xenografts. Immunohistochemistry staining of CK7 (**A, D**), MUC1 (**B, E**) and Hematoxylin and Eosin staining (H&E) of orthotopic xenograft (**C**). Scale bars, 100 μm.

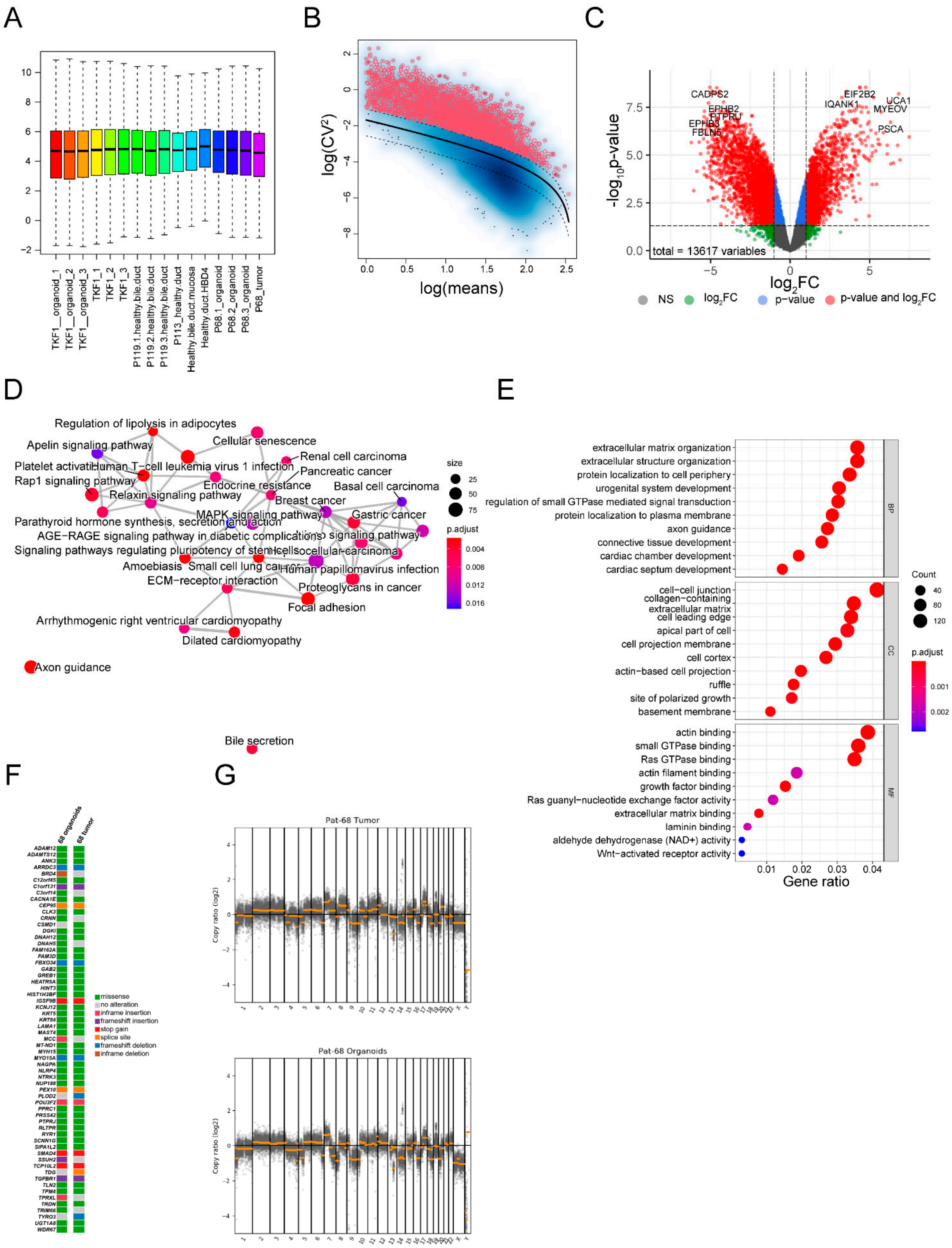

Figure S3. Patient-derived organoids recapitulate the patient features. (**A**) Box plot of all high-throughput RNA sequencing samples after the processing and normalization; (**B**) The scatter plot of variable genes, each red dot represent one significant variable gene, there are 2770 in total; (**C**) The volcano plot reflects the existing significant different expression features between cholangiocarcinoma and healthy tissue. (**D**) The network of significantly enriched pathways in the P68

primary tumor. (**E**) The dot plot demonstrates the most highly enriched gene ontology terms in P68 malignant tumor. (**F**) Non-synonymous variants in P68 organoids and the parental tumor. (**G**) Copy number variant comparison between P68 tumor and corresponding organoids. Abbreviations: CV, coefficient of variation; FC, fold change; NS, not significant.

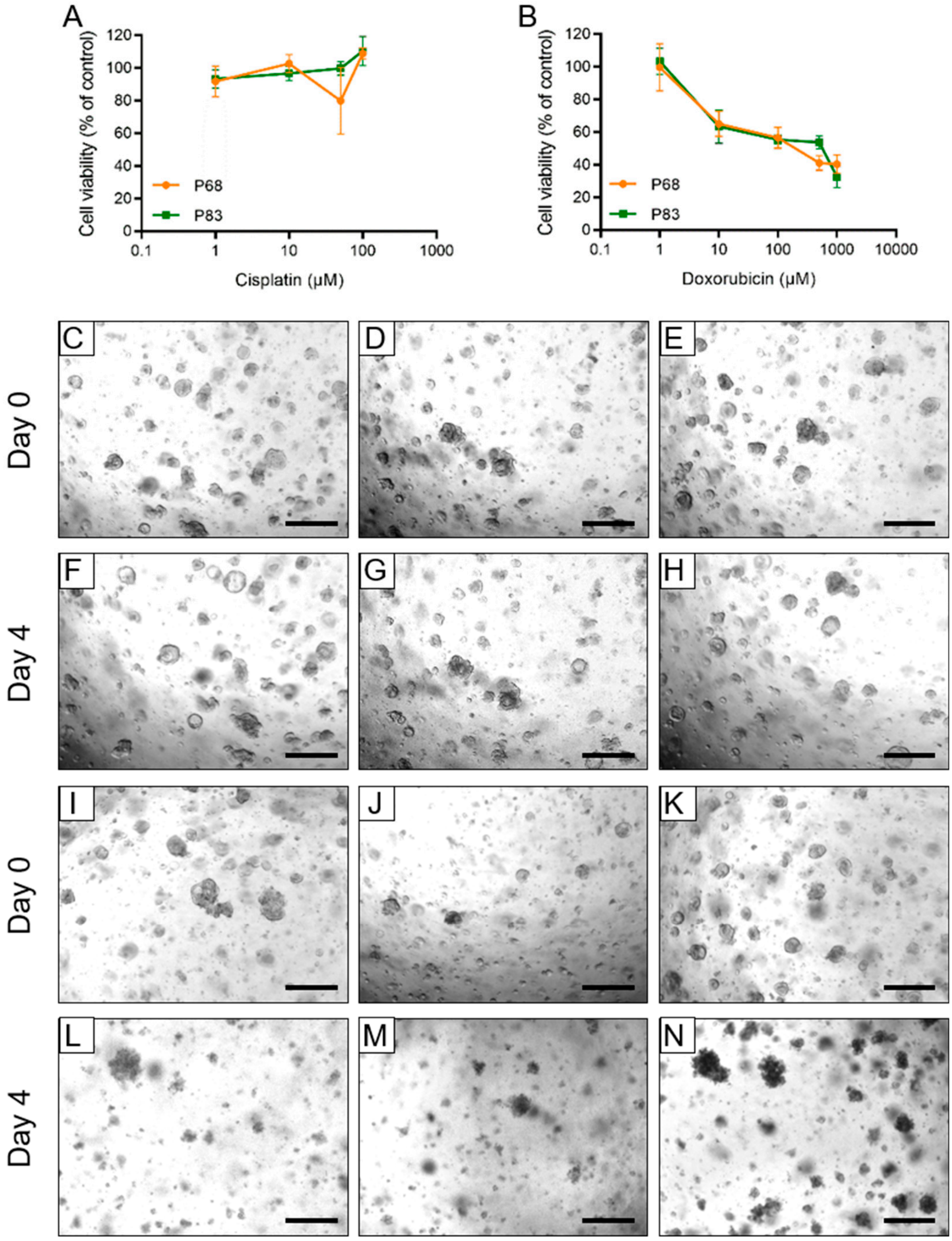

Figure S4. Drug test on patient-derived organoids. Dose responses of tumor organoids (n = 3) measured after 4 days with (**A**) Cisplatin, and (**B**) Doxorubicin. Microscopic assessment of P83 cholangiocellular tumor organoids before and after Doxorubicin treatment. Day 0 corresponds to the time at which the organoids were treated with doxorubicin. Control group (**C**, **F**), organoids treated with different drug concentration: 1 μmol (**D**, **G**), 10 μmol (**E**, **H**), 100 μmol (**I**, **L**), 500 μmol (**J**, **M**), and 1mmol (**K**, **N**); Magnification: 5x; Scale bar, 200 μm.

References

1. Barker, N.; Huch, M.; Kujala, P.; van de Wetering, M.; Snippert, H.J.; van Es, J.H.; Sato, T.; Stange, D.E.; Begthel, H.; van den Born, M.; et al. Lgr5(+ve) Stem Cells Drive Self-Renewal in the Stomach and Build Long-Lived Gastric Units in Vitro. *Cell Stem Cell* **2010**, *6*, 25–36, doi:10.1016/j.stem.2009.11.013.
2. Bolger, A.M.; Lohse, M.; Usadel, B. Trimmomatic: A Flexible Trimmer for Illumina Sequence Data. *Bioinformatics* **2014**, *30*, 2114–2120, doi:10.1093/bioinformatics/btu170.
3. Dobin, A.; Davis, C.A.; Schlesinger, F.; Drenkow, J.; Zaleski, C.; Jha, S.; Batut, P.; Chaisson, M.; Gingeras, T.R. STAR: Ultrafast Universal RNA-Seq Aligner. *Bioinformatics* **2013**, *29*, 15–21, doi:10.1093/bioinformatics/bts635.
4. Liao, Y.; Smyth, G.K.; Shi, W. The Subread Aligner: Fast, Accurate and Scalable Read Mapping by Seed-and-Vote. *Nucleic Acids Res* **2013**, *41*, e108, doi:10.1093/nar/gkt214.
5. Love, M.I.; Huber, W.; Anders, S. Moderated Estimation of Fold Change and Dispersion for RNA-Seq Data with DESeq2. *Genome Biol.* **2014**, *15*, 550, doi:10.1186/s13059-014-0550-8.
6. Yu, G.; Wang, L.-G.; Han, Y.; He, Q.-Y. ClusterProfiler: An R Package for Comparing Biological Themes among Gene Clusters. *OMICS* **2012**, *16*, 284–287, doi:10.1089/omi.2011.0118.
7. Ashburner, M.; Ball, C.A.; Blake, J.A.; Botstein, D.; Butler, H.; Cherry, J.M.; Davis, A.P.; Dolinski, K.; Dwight, S.S.; Eppig, J.T.; et al. Gene Ontology: Tool for the Unification of Biology. The Gene Ontology Consortium. *Nat. Genet.* **2000**, *25*, 25–29, doi:10.1038/75556.
8. Kanehisa, M. Toward Understanding the Origin and Evolution of Cellular Organisms. *Protein Sci.* **2019**, *28*, 1947–1951, doi:10.1002/pro.3715.
9. Talevich, E.; Shain, A.H.; Botton, T.; Bastian, B.C. CNVkit: Genome-Wide Copy Number Detection and Visualization from Targeted DNA Sequencing. *PLoS Comput Biol* **2016**, *12*, e1004873, doi:10.1371/journal.pcbi.1004873.
