# Supplementary material for: Patient-Derived Organoids of Cholangiocarcinoma
Source: Int J Mol Sci. 2021 Aug 12;22(16):8675. doi: 10.3390/ijms22168675 (PMC8395494; doi:10.3390/ijms22168675)
Supplement: Supplementary file 1 [file ijms-22-08675-s001.zip › Informed Consent Statement.pdf]

Universitätsklinikum Carl Gustav Carus
an der Technischen Universität Dresden
Anstalt öffentlichen Rechts

Einwilligungserklärung

Aufbewahrung und Weiterverwendung von Gewebeproben und Körperflüssigkeiten in der Gewebebank des Universitätsklinikums Carl Gustav Carus Dresden

---

Name, Vorname :

Geburtsdatum: Alter:

Kontaktadresse :

Fachabteilung:

PID :

Fall- Nr.:

Aufnahmedatum:

Station:

Ich erkläre, dass ich diese Einwilligungserklärung und die zugehörige Patienteninformation zur Aufbewahrung und Weiterverwendung von Material in der Gewebebank des Universitätsklinikums Carl Gustav Carus erhalten, gelesen und verstanden habe. **Ich hatte Gelegenheit, offene Fragen mit meinen behandelnden Ärzten zu klären.**

Ich erkläre meine Einwilligung, dass meine Proben (z.B. Blut oder Gewebe) für die wissenschaftliche Erforschung von Erkrankungen (einschließlich genetischer Untersuchungen) aufbewahrt und verwendet werden können, soweit sie nicht mehr für Routineuntersuchungen benötigt werden.

Ich bin damit einverstanden, dass meine Proben unbefristet aufbewahrt werden.

Mir ist bewusst, dass ich über die Ergebnisse der wissenschaftlichen Untersuchungen nicht informiert werde. Falls sich aus Ergebnissen unmittelbare medizinische Handlungsempfehlungen für meinen individuellen Krankheitsverlauf ergeben könnten, möchte ich informiert werden:

☐ Ja ☐ Nein

(Ich bin mir über mögliche Offenbarungspflichten, z.B. gegenüber Versicherungen, die daraus folgen können, bewusst)

Ich verzichte auf eine Entgeltzahlung und patentrechtliche oder vergleichbare Ansprüche dafür, dass ich die Proben der Forschung zur Verfügung stelle. Die Proben werden nicht veräußert oder direkt kommerziell genutzt, können aber unter Wahrung der Anonymität industriellen Kooperationspartnern zur Verfügung gestellt werden.

Ich weiß, dass ich jederzeit meine Einwilligung, ohne Angabe von Gründen, widerrufen kann, ohne dass dies für mich nachteilige Folgen hat. Hierzu kann ich mich an das Institut für Pathologie des Universitätsklinikums Dresden, Schubertstrasse 15, 01309 Dresden, Telefon 0351-458 3000 wenden. Meine Proben und die über mich gespeicherten Daten werden im Fall des Widerrufs vernichtet.

Universitätsklinikum Carl Gustav Carus
an der Technischen Universität Dresden

Anstalt öffentlichen Rechts

Ich erkläre mich damit einverstanden, dass im Rahmen der Aufbewahrung von Material in der Gewebebank des Universitätsklinikums erhobene Daten/Krankheitsdaten auf Fragebögen und elektronischen Datenträgern aufgezeichnet und in verschlüsselter Form (ohne Namens- und Initialennennung) weitergegeben werden an

- **mögliche Auftraggeber von Studien zur wissenschaftlichen Auswertung (nach einer entsprechenden Prüfung durch die Ethikkommission der Medizinischen Fakultät der Technischen Universität Dresden und des Beirates der Gewebebank);**

- **an die Ethikkommission der Medizinischen Fakultät der Technischen Universität Dresden, soweit dies zur Überprüfung der ordnungsgemäßen Durchführung der Studien erforderlich ist.**

Ich bin damit einverstanden, dass einer der Forscher mit mir oder meinem Hausarzt in Zukunft Kontakt aufnehmen kann, ohne dass für mich eine Auskunftspflicht hieraus erwächst:

☐ Ja ☐ Nein

Ort	Datum	Unterschrift des Patienten bzw . des gesetzlichen Vertreters

Ort	Datum	Unterschrift Universitätsklinikum

Universitätsklinikum Carl Gustav Carus
an der Technischen Universität Dresden
Anstalt öffentlichen Rechts

Patienteninformation

Aufbewahrung und Weiterverwendung von Gewebeproben und Körperflüssigkeiten in der Gewebebank des Universitätsklinikums Carl Gustav Carus Dresden

Sehr geehrte Patientin, sehr geehrter Patient,

bei Ihnen ist eine diagnostische oder operative Maßnahme aus medizinischer Indikation zur Sicherung der Diagnose bzw. zur Behandlung Ihrer Erkrankung notwendig. Dabei werden Körpergewebe sowie Köperflüssigkeiten wie z.B. Blut für die notwendigen Untersuchungen entnommen. Häufig bleibt Restmaterial übrig. Für wissenschaftliche Untersuchungen zum besseren Verständnis von Erkrankungen ist dieses Material von größter Bedeutung. Wir bitten Sie deshalb um Ihre Zustimmung zur Aufbewahrung und Weiterverwendung dieses Materials zu Forschungszwecken.

Die wissenschaftlichen Untersuchungen betreffen ausschließlich Fragestellungen der Forschung, die dazu beitragen sollen, künftig Diagnosestellungen sicherer zu machen, dass das biologische Verständnis von Erkrankungen verbessert wird und eventuell neue Ansätze zur Behandlung gefunden werden können. Erkranktes Gewebe beinhaltet die gesamte Information über genetische und andere Veränderungen, welche die Erkrankung verursacht und ihren Verlauf bestimmt haben.

Durch die Aufbewahrung würden auch zu einem späteren Zeitpunkt nach dann neuesten wissenschaftlichen Erkenntnissen Untersuchungen an den Geweben durchgeführt werden. Das Material würde hierzu ohne eine zeitliche Begrenzung in der Gewebebank des Universitätsklinikums gelagert werden.

Parallel zur Aufbewahrung des Materials werden personenbezogene Daten erhoben und auf Datenbanken pseudonymisiert gespeichert, das heißt, dass Ihre Daten und das von Ihnen zur Verfügung gestellte Material einer Nummer zugeordnet werden. Eine Verknüpfung der Nummer mit Ihrem Namen ist jeweils nur Ihren behandelnden Ärzten möglich. Alle Personen, die Einblick in die Daten haben, sind zur Wahrung des Datengeheimnisses und der ärztlichen Schweigepflicht verpflichtet.

Das Gewebe und andere Material sowie klinische Daten werden dabei **anonymisiert** an andere Forscher (evtl. auch industrielle Forschungslabore) zu Entwicklungs- und Forschungszwecken zur Untersuchung weitergegeben. Dies erfolgt nur, nachdem die entsprechenden Untersuchungen von der zuständigen Ethikkommission der Medizinischen Fakultät der Technischen Universität Dresden genehmigt wurden.

Eine unmittelbare kommerzielle Nutzung des von Ihnen zur Verfügung gestellten Materials erfolgt nicht. Umgekehrt verzichten Sie durch die Einwilligung zur Materialaufbewahrung und -weiterverwendung auf etwaige rechtliche Ansprüche, soweit diese aus einem Erkenntnisgewinn aus Forschungsprojekten resultieren, die unter Verwendung Ihres Materials erwachsen sollten.

Eine Information an Sie über etwaige wissenschaftliche Erkenntnisse, die unter anderem aus dem von Ihnen zur Verfügung gestellten Material gewonnen werden, ist nicht vorgesehen. Falls Sie Auskunft über mögliche individuelle Ergebnisse wünschen, insbesondere bei genetischen Untersuchungen, müssen Sie sich über mögliche Offenbarungspflichten bewusst sein, die für Sie daraus folgen können (z.B. gegenüber Versicherungen).

Sie haben jederzeit die Möglichkeit, Ihre Einwilligung zur Aufbewahrung und Weiterverwendung der Proben für wissenschaftliche Zwecke zurückzunehmen. Hierzu wenden Sie sich an das Institut für Pathologie des Universitätsklinikums Dresden, Schubertstrasse 15, 01309 Dresden, Telefon 0351-458 3000. Die zu wissenschaftlichen Zwecken gelagerten Materialien würden dann vernichtet werden.

Im Übrigen möchten wir betonen, dass Ihre Ablehnung oder Zustimmung zur Materialaufbewahrung Ihre Behandlung in keiner Form beeinflusst.

Die Möglichkeit, durch Gewebebanken Krankheiten, ihre Entstehung und Behandlung erforschen zu können, kommt allen Betroffenen zu Gute. Wir bitten Sie deshalb um Unterstützung für den Aufbau einer Gewebebank im Universitätsklinikum Carl Gustav Carus Dresden an der Technischen Universität Dresden.

Nutzen Sie das Gespräch mit Ihren behandelnden Ärzten, falls Sie Rückfragen haben.

Prof. Dr. med. G. Baretton
Direktor des Institutes für Pathologie

Wir danken Ihnen für Ihre Mühe und Unterstützung und wünschen Ihnen eine gute Genesung!
